# Factors influencing postoperative atrial fibrillation in patients undergoing on-pump coronary artery bypass grafting, single center experience

**DOI:** 10.1186/s13019-017-0609-1

**Published:** 2017-05-23

**Authors:** Mohamed F. Ismail, Ahmed F. El-mahrouk, Tamer H. Hamouda, Hanan Radwan, Ali Haneef, Ahmed A. Jamjoom

**Affiliations:** 1 Cardiothoracic Surgery Department, Faculty of medicine Mansoura University, Mansoura, Egypt; 2 Cardio-Thoracic Surgery Department, Faculty of medicine Tanta University, Tanta, Egypt; 3 Cardiothoracic Surgery Department, Faculty of Medicine Benha University, Benha, Egypt; 4 Cardiology Department, Faculty of Medicine Zagazig University, Zagazig, Egypt; 5 King Faisal Specialist Hospital and Research Center, Jeddah, Saudi Arabia

**Keywords:** CABG, Atrial fibrillation, Predictors, Postoperative outcome

## Abstract

**Background:**

The reported incidence of AF after CABG surgery varies from 20 to 40%, with the arrhythmia usually occurring between second and fourth postoperative days. Postoperative AF after CABG was associated with greater in-hospital mortality and worse survival at long-term follow-up. Therefore, intensive attention has focused on the prevention of AF in high-risk patients. Many perioperative factors have been suggested to increase the incidence of postoperative AF after conventional CABG. In this study we are trying to examine some of these risk factors as predictors for Post-operative AF in our patients. In this study, our aim was to identify the perioperative predictors of AF in our patients who underwent Coronary Artery Bypass Grafting.

**Methods:**

Our Patients were divided into two groups; Group A included patients who did not develop PO AF (168 patients) and Group B patients who developed PO AF (84 patients). Perioperative Data, including gender, age, demographic variables and postoperative morbidity and mortality were extracted from the medical records.

**Results:**

This retrospective cohort study was conducted on 252 consecutive adult patients underwent CABG, in King Faisal Specialist Hospital and Research Center in Jeddah, Saudi Arabia. The mean age for patients with PO AF was 65 years (*P* = .0001). Eight-three patients (49.4%) were diabetics in group A and 56 patients (66.7%) in group B (*P* = .0001). Patients who developed POAF had a lower ejection fraction (44.8 ± 5.7%) (*P* = .0001), diastolic dysfunction (*P* = .0001), Larger Left atrial volume (*P* = .0001). Bleeding requiring re-opening for exploration and Postoperative shock were identified as significant predictors for POAF. Multivariate logistic regression (odds ratio, ±95% CI, P value) was performed to identify the effect of age, preoperative heart rate, ejection fraction, postoperative bleeding, Shock, ventilator time, Sensitivity was 89.5%, specificity was 94.6%, positive predictive value was 89.5%, and negative predictive value was 94.6%.

**Conclusion:**

In our study, advanced age, enlarged LA volume, low ejection fraction, combined surgeries and prolonged ventilation time were found to be predictors of atrial fibrillations after coronary artery bypass grafting.

## Background

Atrial fibrillation (AF) is a supraventricular tachyarrhythmia characterized by uncoordinated atrial activation with subsequent deterioration of mechanical function [1, 2]. The incidence of AF after CABG surgery varies from 20 to 40%, with the arrhythmia usually occurring between the second and fourth postoperative days [3–5]. Peak incidence was found on postoperative day two [1].

This incidence far exceeds its reported prevalence in the general population and patients with atherosclerotic coronary artery disease (CAD) [3]. Similarly, it is significantly more than the reported incidence of AF after major non-cardiac surgery regardless of CAD status [6]. The pathophysiological mechanisms responsible for the high rate of AF after cardiac surgery in general and after CABG surgery, in particular, remain unclear.

Although this arrhythmia is usually benigne and self-limiting, it may result in hemodynamic instability and increases the risk of congestive heart failure (CHF), longer ICU stays and longer hospital stay, hence increased healthcare costs [3, 5].

Thromboembolic events are also a major complication; Stroke was seen in 2% of CABG patients, 37% of whom had preceding AF. Postoperative AF after CABG was associated with greater in-hospital mortality and worse survival at long-term follow-up (4–5 years) [7]. Therefore, intensive attention has focused on the prevention of AF in high-risk patients. Multiple investigations have attempted to identify the demographic risk factors and the predictors of postoperative AF. Many perioperative factors have been suggested to increase the incidence of postoperative AF after conventional CABG, such as advanced age, hypertension, withdrawal of b-blocker drug, RCA stenosis, respiratory complications, and bleeding [4, 5, 8].

Poor cardioplegic protection of the atria during Cardiopulmonary bypass and myocardial ischemia have been reported to increase the incidence of postoperative AF [4].

In this study, our aim was to study some of the factors Influencing Postoperative Atrial Fibrillation in Patients Undergoing On-pump in our center.

## Methods

In this retrospective study 252 consecutive adult patients underwent on-pump CABG , between November 2013 and December 2015, in King Faisal Specialist Hospital and Research Center in Jeddah, Saudi Arabia. The protocol of the study was approved by the Local Ethics Committee.

### Clinical data collection

Our Patients were divided into two groups; Group A included patients who did not develop post operative AF (168 patients) and Group B patients who developed postoperative AF (84 patients). Data, including gender, age, demographic variables and postoperative morbidity and mortality were extracted from the medical records. All patients transferred to the telemetry floor for monitoring after discharging from ICU during their hospital stay.

Preoperative variables were: age, sex, smoking, hypertension, dyslipidemia, Diabetes Mellitus, Peripheral Vascular Disease (PVD), preoperative medications, Chronic Obstructive Pulmonary Disease (COPD), old Myocardial infarction (MI). Preoperative hemodynamics (Blood pressure, Heart rate), Preoperative creatinine or hemodialysis and preoperative echo data. The Euro- SCORE was recorded for all patients.

Operative Data included: The type of surgery performed, cross clamp and total bypass time, the lowest body temperature, the number of grafts. Intraoperative complications (arrhythmias, hemodynamic instability, intra-aortic balloon, acute myocardial infarction, bleeding). The use of inotropes (noradrenaline, dopamine, dobutamine, nitroglycerin, sodium nitroprusside). Blood transfusion and blood products (platelets, packed red blood cells, and fresh frozen plasma).

The postoperative data included: Ventilation time in hours, Intensive care unit stay, hospital stay, mortality, shock, stroke, infections, renal failure and bleeding.

### Exclusion criteria

- Chronic Atrial Fibrillation.
- Documented Cardiac Arrhythmias
- Antiarrhythmic drugs other than beta-blockers.
- Previously implanted pacemaker.
- Uncontrolled heart failure.
- Emergency surgeries.
- Redo-surgery.
- Combined surgeries.
- Off pump CABG.

### Definitions

- Post Operative Atrial fibrilation ( POAF): New onset of Atrial Fibrillation occurring in the immediate postoperative period despite correction of electrolyte deficit and needed electrical or pharmacological cardioversion.
- Cardiogenic shock was defined as “hypotension (a systolic blood pressure < 90 mmHg) and/or a cardiac index < 2.0 for at least 30 min, or the need for supportive measures to maintain a systolic pressure > = 90 mmHg or a cardiac index > 2.0”.
- Postoperative renal failure was defined as an increased plasma creatinine ≥ 2 associated with urine production less than 0.5 mL/kg/hour ≥ 12 h, or patients requiring dialysis.
- Bleeding requiring re-opening for exploration was defined as the need for chest reopening in the presence of more than 500 mL of blood from chest tubes within the first hour, more than 400 mL within the second hour, more than 300 mL within the third hour, or total bleeding greater than 1000 mL within the fourth hour.

### Statistical analysis

was conducted using the SPSS19.0 statistical software package (SPSS, Inc., Chicago, IL). All *P* values < 0.05 were considered statistically significant. Quantitative variables are expressed as a mean ± standard deviation, and the qualitative values as percentages. Univariate analysis, of pre-, intra- and post-operative variables with the occurrence of postoperative AF using the unpaired t-test to compare measurement data and Fisher’s exact test to compare enumeration data, was performed to assess statistically significant variables. Stepwise multivariate logistic regression was also performed.

## Results

Two hundred and fifty-two consecutive patients were enrolled in our study; Male-gender was (116) 69% in group A and (52) 61.9% in group B. Age ranged from 39 to 87 years; with a median age of 65 years. Patients with age more than 70 years were 61%; The mean age for patients with postoperative AF was 65 years compared with 54 for patients without (*P* = .0001). The incidence of POAF was directly proportional to the age, where the incidence was 7% in patients less than 60 years old, 18% between 60 and 70 years and reached up to 42% for patients above the age of 70 years.

Eighty-three patients (49.4%) had diabetes in group A and 56 patients (66.7%) in group B (*P* = .0001). Other preoperative characteristics were matching in both group; no significant difference was observed between the two groups about gender, Smoking, dyslipidemia, previous old MI, peripheral vascular disease, medical therapy, early cerebro-vascular accident chronic obstructive lung disease or clinical presentation of CAD. Demographic, clinical, and perioperative variables are illustrated in Table 1.

**Table 1:** Preoperative characteristics

Variable	No AF (*n* = 168)	AF (*n* = 84)	*P* value
Age	54.69 ± 15.18	65.36 ± 9.96	<0.001
Gender male	116 (69%)	52 (61.9%)	0.276
Hypertension	118 (70.2%)	57 (67.9%)	0.554
Diabetes mellitus	83 (49.4%)	56 (66.7%)	0.001
Dyslipidemia	120 (71.4%)	64 (76.2%)	0.082
Smoking	70 (41.7%)	26 (30.9%)	0.063
Old MI	54 (32.1%)	33 (39.3%)	0.158
Peripheral Vascular Disease	10 (5.9%)	6 (7.1%)	0.476
COPD	15 (9.3%)	10 (11.9%)	0.231
Old CVA	14 (8.4%)	8 (9.5%)	0.438
Systolic blood pressure	123.8 ± 19.2	123.9 ± 28.1	0.994
Preoperative heart rate	75.9 ± 11.3	80.1 ± 17.1	0.042
Preoperative creatinine	136.42 ± 21.49	113.19 ± 14.24	0.496
Hemodialysis	12 (7.1%)	7 (8.3%)	0.482
Beta blockers	157 (93.5%)	77 (91.7%)	0.462
Calcium channel blockers	46 (27.4%)	15 (17.9%)	0.076
Digoxin	24 (14.3%)	10 (11.9%)	0.322
Diuretics	27 (16.1%)	11 (13.1%)	0.305
Statins	140 (83.3%)	73 (86.9%)	0.302
ACEI	110 (65.5%)	65 (77.4%)	0.186
Thyroxin	14 (8.4%)	10 (11.9%)	0.182
Diseased vessels	2.26 ± 1.2	2.51 ± 0.9	0.072
Left main >50%	70 (41.7%)	34 (40.5%)	0.480
LAD > 70%	133 (79.2%)	69 (82.1%)	0.122
LCX >70%	120 (71.4%)	67 (79.8%)	0.086
RCA >70%	112 (66.7%)	62 (73.8%)	0.140
RI >70%	35 (20.8%)	11 (13.1%)	0.085

Echo data in Table 2, shows that patients who developed postoperative AF had a lower ejection fraction (44.8%) compared to those with no Postoperative AF (56.7%) (*P* = .0001). Furthermore, patients with postoperative AF had more diastolic dysfunction (*P* = .0001), Larger Left atrial volume (35.9 mm) compared to (30.4 mm) in the other group (*P* = .0001). Preoperative serum creatinine and the number of patients with end stage renal disease on hemodialysis were not significantly different.

**Table 2:** Preoperative echo data

Preoperative Echo data	No AF (*n* = 168)	AF (*n* = 84)	*P* value
Diastolic function grade 0	117 (69.6%)	6 (7.1%)	<0.001
Diastolic function grade 1	37 (22.1%)	13 (15.5%)	0.144
Diastolic function grade 2	9 (5.4%)	34 (40.5%)	<0.001
Diastolic function grade 3	5 (2.9%)	31 (36.9%)	<0.001
LA volume	30.45 ± 3.7	35.91 ± 1.2	<0.001
EA ratio	1.19 ± 0.29	1.67 ± 0.52	<0.001
D time	197.19 ± 39.27	168.37 ± 45.68	<0.001
LV EF	56.7 ± 6.2	44.8 ± 5.7	<0.001

In patients with CAD, there was no statistical difference between both groups regarding diseased vessels nor the number of grafts performed. We found no significate difference between both groups regarding cross clamp time, total bypass time or the degree of hypothermia Table 3. However, postoperative Data revealed that patients who developed postoperative AF had more bleeding requiring re-opening for exploration 8 (9.5%) patients compared to 2 (1.2%) patients in the no AF group (*P* = .0003). The postoperative shock was also higher in the postoperative AF group 11 (13.1%) patients in comparison to no AF group 1 (0.6%) patients with P value < 0.001. Similarly, the ICU stay, ventilation time, and in hospital stay was statistically longer in the AF group than the other group. Mortality was 1 patient (0.6%) in group A and 1 patient (1.2%) in group B, *P* = 0.555, Table 4.

**Table 3:** Operative characteristics

Intraoperative	No AF (*n* = 168)	AF (*n* = 84)	*P* value
CPB time	115.8 ± 45.4	120.4 ± 48.12	0.562
Cross clamp time	91.5 ± 43.7	95.8 ± 41.2	0.285
Lower temperature	33.2 ± 4.01	33.5 ± 2.08	0.506
IABP	11 (6.5%)	7 (8.3%)	0.408
Urgency	18 (10.7%)’	13 (15.4%)	0.142

**Table 4:** Postoperative characteristics

Variable	No AF (*n* = 168)	AF (*n* = 84)	*P* value
Ventilation time (Hs)	16.1 ± 12.12	23 ± 15.1	0.003
Potassium (mmol/l)	4.56 ± 1.12	3.47 ± 0.84	0.044
Phosphorus (mmol/l)	1.15 ± 0.49	0.67 ± 0.37	0.095
Magnesium (mmol/l)	1.16 ± 0.47	0.58 ± 0.29	0.032
Infection	2 (1.2%)	3 (3.5%)	0.168
Stroke	3 (1.8%)	4 (4.8%)	0.125
Bleeding	2 (1.2%)	8 (9.5%)	0.0003
Shock	1 (0.6%)	11 (13.1%)	<0.001
Renal failure	25 (14.9%)	15 (17.8%)	0.362
ICU stay	3.8 ± 1.48	5.58 ± 2.97	0.019
Length of stay	11.57 ± 8.15	32.21 ± 13.52	0.023
Mortality	1 (0.6%)	1 (1.2%)	0.555

Postoperative Serum level of electrolytes Potassium (normal value 3.5–5 mmol/l), Phosphorus (normal value 0.8–1.5 mmol/l) Magnesium (normal value 0.75–0.95 mmol/l)

Multivariate logistic regression (odds ratio, ±95% CI, *P* value) was performed to identify the effect of age, [OR = 2; CI, 1.067 to 1.252; *P* = .001) diabetes, (OR = 3.5; CI, 0.002 to 0.322; *P* = .0001), preoperative heart rate (OR = 1.7; CI, 0.981 to 1.068; *P* = .004), ejection fraction (OR = 3.9; CI, 0.579 to 0.809; *P* = .01), postoperative bleeding, (OR = 2; CI, 0.00 to 4.633; *P* = .003), Shock (OR = 3.5; CI, 0.02 to 4.64; *P* < .001 ventilator time, (OR = 3.2; CI, 1.012 to 1.82; *P* = .003). Sensitivity was 89.5%, specificity was 94.6%, positive predictive value was 89.5%, and negative predictive value was 94.6%. Of these predictor variables: age, diabetes mellitus, LA volume, ejection fraction, E/A ratio and ventilation time were found to be significant.

## Discussion

Atrial fibrillation is considered the commonest arrhythmia after cardiac surgery. It has an impact on the clinical situation, hemodynamic stability, thromboembolic events, the hospital stay as well as a direct impact on hospital cost [4]. AF is a main contributing factor for increasing postoperative morbidity and mortality [3, 4].

Despite advances in techniques of surgery, CPB, and cardioplegic arrest, the occurence of post cardiac surgeries AF has significantly increased [9]. This happened because of patients, tend to be older and iller, with a great frailty index, and this high-risk subgroup is more liable for complications. It is also of note that, the use of telemetry (continuous ECG monitoring) has improved its early detection [10]. Abundant studies were implemented to identify the predictors of atrial fibrillation for the creation of an atrial fibrillation risk score. In this retrospective research, we are trying to investigate the perioperative predictors of AF for our patients with on-pump CABG [10, 11].

The increasing incidence of AF after heart procedures is yet to be elucidated; many factors were contemplated to work in the pathogenesis of postoperative AF like changes of the atria (increased fibrosis and atrial dilatation). These changes are thought because of age, mechanical damage, volume overload, intraoperative atrial ischemia, electrolyte imbalances, hypertension and pericardial lesions (pericarditis) [4]. Additionally, the significant rise in the perioperative sympathetic tone, plays a major role in the development of AF. [9] All the above mentioned factors when occurring alone or in different combinations result in a high likelihood of development of POAF.

Many Perioperative variables have been proposed as predictors that increase the appearance of postoperative AF after cardiac surgery like advanced age [10], hypertension [10], β-blocker drug withdrawal [12], respiratory complications [5], right coronary artery stenosis [8], and bleeding [13].

Age has been a convenient independent predictor of AF in general [14]. Moreover, increased age is the most frequently recognized risk factors for the incidence of AF after CABG. This higher occurence of AF with older age could be attributed to Age-related comorbidities [4]. This has long been explained by the fact that aging causes degenerative changes in the atrium as well as changes in atrial physiology. Amar and colleges, 2002, concluded these changes as “shorter effective refractoriness, delayed SA and AV nodal conduction times, atrial stiffening, and splitting of the atrial excitation waveform caused by the pectinated trabeculae” [15]. Additionally, Trauma to sympatho-vagal fibers of the cardiac plexus during surgery may be contributed to the aging of patients that can lead to POAF [16].

In the current study, 96 patients (35%) had POAF. POAF was higher in patients older than 60 years of age; age has been consistent with an increased incidence of POAF. Moreover, this frequency consistently increases with advancement of patient age at the time of surgery. These results were in agreement with several series that suggest an impact of POAF ranging from 25 to 53% [4].

In this study, POAF increased with the advancement of age, from 7% in those patients less than 60 years old to 18% between 60 and 70 years and that incidence reached up to 42% for patients above the age of 70 years. Mathew et al., 2004 [2], stated that “every 10-year increase is associated with a 75% increase in the odds of developing AF”.

Besides the increasing age, we identified: Diabetes Mellitus, as independent predictors of POAF. Clinical evidence suggests that DM and AF are strongly interconnected. A study carried out in 2005 by Mohaved et al., reported that: “incidence of AF was significantly higher in patients with DM, compared to a control group.” They also mentioned that: “atrial flutter occurred in 4% of DM patients vs. 2.5% in the control group” [17]. Furthermore, in their study, Pathak et al., elucidate that DM is closely related to metabolic syndrome, which includes obesity, a one of the established risk factor for POAF [18]. Framingham Heart group demonstrated that “risk factors for cardiovascular disease including Diabetes Mellitus, predispose to AF. Diabetes Mellitus gave an odds ratio of 1.4 and 1.6 for men and women respectively, for developing AF” [19].

It is of note that these results came from patients not subjected to cardiac surgery, and adding the factors associated with Cardiac surgery would increase the incidence of POAF [4, 9, 15].

In our current series, Preoperative variables, including large left Atrial volume, depressed left ventricular function (both systolic and diastolic) and high preoperative heart rate, have been associated with the increased incidence of POA. Enlarged LA size is consistently reproducible predictors for POAF. The influence of enlarged left atrial on POAF has been demonstrated with CABG in several studies [20]. However, other previous reports [2, 11, 21], abandon the correlation of the size of left atrium and AF, assuming that dysfunctional left atrial is distinct of atrial remodeling. That may atributed to the younger age of their population in comparison to ours. Increasing collagen of the left atrium in aging populations and the other aging biology can also have a role in the appearance of AF.

In our series, there was increased incidence of POAF with elevated A/E ratio. It could be explained by, chronically high filling pressures that increase the stretch of left atrial myocyte, producing an elevated left atrial effective refractory period dispersion that leads to the development of AF [22].

Echo data also illustrated a considerable association between POAF and diastolic dysfunction; That goes in line with the Cardiovascular Health Study; this mega study followed 4480 subjects for a mean of 12.1 years examining diastolic dysfunction and AF, with 1219 cases (27.2%) of incident AF [23]. This study found that the LA dimension, the trans-mitral E-wave velocity, and the trans-mitral A-wave velocity-time integral were significantly and independently correlating with the incidence of POAF, and may be used in the prediction of AF in elderly. It advocates the assumption of the mechanism for risk factors like hypertension to participate in the incidence of AF, is through diastolic dysfunction [23].

Again, these data were extracted from participants not subjected to Coronary surgery. Hence, intraoperative atrial ischemia, electrolyte imbalances, pericardial lesions and the significant rise in the perioperative sympathetic tone, associated with this surgery, would prominently increase the incidence of POAF [4, 9, 15].

Postoperative data in this cohort, showed no difference between both groups regarding the electrolytes level, this can be attributed to the the higher tendency for correcting any electrolytes deficits.

It is of worth-mentioning that more bleeding requiring re-opening for exploration (9.5%) and patients with cardiogenic shock (13.1%) were found in POAF group this can be explained by the delay in resuming Beta-blocker in these patients due to the hemodynamic condition of these patients.

Our data demonstrated that patients with a lower ejection fraction are more liable to develope POAF. Kannell and colleges, 1982, stated that: “poor left ventricular function and congestive heart failure are associated with a greater risk for the development of AF in the general population” [24]. Additionally, the reduced left ventricular function is a risk factor for the development of AF after cardiac surgery [4]. This impairment correlates with higher left ventricular preload (as assessed by A/E ratio) [25].

In the present study, it was difficult to distinguish a significant relation between cross-clamp time, cardiopulmonary bypass time, and the incidence of POAF. This suggestion goes with other reports [5]. However, in contradiction with that, many studies showed that prolonged bypass time and cross-clamp times to be independent predictors for POAF [26]. They postulated that Inadequate atrial protection with their cardioplegia techniques and extended periods of ischemia led to atrial ischemia and probably triggered the development of AF in vulnerable patients [27].

We also showed that patients with POAF were significantly had prolonged length of intensive care unit stay and inhospital stay time. Consequently, this will increase the cost of surgery [28]. Prophylactic measures targeting patients at high risk of POAF, including pharmacologic strategies will significantly reduce the associated costs [29].

## Conclusion

In our study, advanced age, enlarged LA volume, low ejection fraction, combined surgeries and prolonged ventilation time were found to be important factors that can increase the incidence of atrial fibrillations after coronary artery bypass grafting with subsequent impact on hemodynamic stability, thromboembolic events and the hospital stay.

Prophylactic measures including, pharmacological strategies as early resumption of beta Blockers and early correction of electrolyte imbalance particularly targeting patients at risk can decrease the financial burden and more importantly decrease postoperative morbidity and mortality.
